# School Refusal in Immigrants and Ethnic Minority Groups: A Qualitative Study of Adolescents' and Young Adults' Experiences

**DOI:** 10.3389/fpsyt.2022.803517

**Published:** 2022-04-11

**Authors:** Camille Brault, Isaiah Thomas, Marie Rose Moro, Laelia Benoit

**Affiliations:** ^1^Etablissement Public de Santé Ville-Evrard, Neuilly sur Marne, Paris, France; ^2^Yale Child Study Center, Yale School of Medicine, New Haven, CT, United States; ^3^Maison des Adolescents—Maison de Solenn, Hôpital Cochin, AP-HP, Paris, France; ^4^University of Paris, Faculty of Psychology, Boulogne-Billancourt, Paris, France; ^5^Inserm U1018, Team DevPsy, CESP, Paris Saclay University, Paris, France

**Keywords:** school refusal, immigrant, youth, school absenteeism, ethnic minority, adolescence, racism, qualitative study

## Abstract

**Background:**

School refusal is one cause of school absenteeism along with truancy, and the two can be difficult to distinguish. School absenteeism behaviors among students in transcultural situations (immigrants or children of immigrants) and from ethnic minority groups are subject to misdiagnosis and decreased access to care. To improve the care provided, this exploratory study addresses the experience of adolescents and young adults engaging in school refusal, from immigrant and ethnic minority groups.

**Methods:**

Sixteen participants between the ages of 16 and 20 years old presenting with school refusal were interviewed for this qualitative study. All participants were either immigrants, children of immigrants, or from an ethnic minority group. We conducted a qualitative analysis based on Interpretative Phenomenological Analysis.

**Results:**

Participants experienced school refusal as a loss of identity and as a failure to achieve what was perceived as parental expectations of success, which triggered feelings of worthlessness, shame, and guilt. The loss of a peer group, namely their classmates, as a result of school absenteeism was experienced as a marginalization from the larger society. Although participants denied having personally experienced racism, some of them recalled their parents experiencing racism at school.

**Conclusion:**

School refusal complicates identity construction, autonomy, and integration into society. For adolescents and young adults from immigrant and ethnic minority backgrounds, it also triggers guilt, transgenerational traumatic memories, and the fear of marginalization. In addition to validated therapies for school refusal, sociological, intersectional, and cross-cultural tools would be a valuable addition to treatment.

## Introduction

For several years, the classification of school absenteeism behaviors has been refined to improve access to appropriate support. Heyne et al. (1) define four categories of “school attendance problems”: school refusal, truancy, academic withdrawal, and academic exclusion. In 2020, 6.8% of students in French public high schools displayed school absenteeism, defined by the French Ministry of Education as at least 2 days of unjustified absences per month (2). Dropping out of school before graduation can have a significant impact on future career prospects, especially in France where college diplomas are greatly valued (3). Accordingly, young people who dropped out of high school without receiving a diploma or who did not enter college after completing high school have a higher unemployment rate in France than in other European countries; in 2017, the unemployment rate of these young people was three times higher than that of students who completed 2 years of college (4).

School absenteeism occurs at higher rates among immigrant students than among non-immigrant students in European countries (5). In 2018, 6.4 million immigrants were living in France, and 7.5 million of those born in France had at least one immigrant parent, together representing 11.2% of the population (6). Students who are immigrants or whose parents are immigrants are more likely to be admitted to vocational programs after secondary school and to complete their education without a diploma (7). In 2012, Chau compared the academic pathways of immigrant students in France of European and non-European origin (8). The latter group had higher rates of class repetition, low academic performance, and cognitive dropout (i.e., the student attends class but is no longer actively involved in learning). Similarly, the academic success of students who are immigrants or whose parents are immigrants varies according to the parents' country of origin (7). These differences are partially, but not entirely, explained by cultural, socio-demographic, and family characteristics. Most likely, they reflect the impact of racism, be it individual or systemic, and subsequent beliefs about ethnic minorities' reduced chances of academic success.

School refusal is one of the four patterns of school absenteeism and accounts for 5% of child and adolescent psychiatry referrals in France (9). School refusal is defined by Heyne et al. (1) as follows: “(1) a young person is reluctant or refuses to attend school, in conjunction with emotional distress that is temporal and indicative of aversion to attendance (e.g., excessive fearfulness, temper tantrums, unhappiness, unexplained physical symptoms) or emotional distress that is chronic and hindering attendance (e.g., depressed mood, sleep disturbance), usually but not necessarily manifesting in absence (e.g., late arrivals; missing whole school days; missing consecutive weeks, months, or years); (2) the young person does not try to hide the associated absence from their parents (e.g., they are at home and the parents are aware of this), and if they previously hid absence, they stopped doing so once the absence was discovered; (3) the young person does not display severe antisocial behavior, beyond resistance to parental attempts to get them to school; and (4) the parents have made reasonable efforts, currently or at an earlier stage in the history of the problem, to ensure attendance at school, and/or the parents express their desire for their child to attend school full-time”. School refusal is frequently associated with other anxious manifestations: phobias regarding transportation; separation anxiety; somatic symptoms (10); behavioral problems, especially in the family setting (11); and depressive symptoms (50%), such as major depression (13.9%) and social withdrawal (12, 13).

A different pattern of school absenteeism is truancy, defined by Heyne et al. (1) as follows: “(1) a young person is absent from school for a whole day or part of the day, or they are at school but absent from the proper location (e.g., in the schoolyard rather than in class); (2) the absence occurs without the permission of school authorities; and (3) the young person typically tries to conceal the absence from their parents”. Teasing apart the differences between school refusal and truancy is challenging. According to Heyne et al., “reasonable parental effort” to get the child to go to class is a specific criterion for school refusal. However, in 2008, Kearney (14) elucidated the lack of consensus regarding metrics of parental involvement. In particular, a student who is an immigrant or the child of immigrants is experiencing a cross-cultural situation: the cultural codes at home differ from those expected in the society's institutions, such as school. Cross-cultural aspects must be considered to better understand the behavior of the student and his or her parents. Kearney argues for stronger parent-teacher collaboration in cross-cultural settings to improve the referrals to care for students who experience school refusal (15).

The obstacles to academic achievement that children from marginalized groups face in France are clearly established. However, research on this population of school refusers remains limited. In one 2015 case study, Benoit et al. (16) described an adolescent immigrant for whom school refusal represented an expression of larger struggles with identity. Since then, Rosenthal et al. (17) showed in a qualitative study with 11 immigrant parents of adolescents that these parents feel overwhelmed by school refusal and that they understand it as a failure to fulfill the immigrant narrative's mandate of success. While immigrant parents initially idealized the school system of their new home country, their experiences of rejection and racism led to disappointment and mistrust. Rosenthal argues that parents' customs and culture, immigration history, and experiences of adversity should be part of assessing school refusal to facilitate access to appropriate care. In a qualitative study of 50 French school professionals, Martin et al. showed that school refusal among immigrant students and students whose parents are immigrants can be misinterpreted as truancy, partly because parental efforts to ensure their child's attendance are not perceived by teachers as such (18). Prejudices about parents' attitudes and behaviors lead teachers to conclude that absenteeism does not reflect anxiety, but rather a lack of interest in school on the part of both the student and the family. Thus, both cultural misunderstandings and racism may delay or prevent referral to care for students experiencing school refusal in a cross-cultural situation or students who are part of marginalized ethnic groups, resulting in them dropping out of school. Such inequalities in health care may result in a self-fulfilling prophecy about the prejudice that immigrant students or children of immigrants are less likely to achieve academic success.

Adding to Benoit's case study (16), this study is the first to explore the views and feelings of several adolescents and young adults in a cross-cultural situation (immigrant students, children of immigrants) and/or from ethnic minority groups, regarding their experience of school refusal.

## Methods

### Study Design

Given that this study is exploratory, the choice of a qualitative method is particularly relevant. This qualitative study has been carried out according to the COREQ criteria (19). Participants were recruited in four child psychiatry departments in Paris and its suburbs (Seine Saint-Denis and Val-d'Oise) (20). Inclusion criteria were: age ≥ 12 years; diagnosis of school refusal; follow-up of at least 6 months; and being born abroad or having at least one parent born abroad (or in the French Caribbean Islands). The definition of school refusal was a combination of Heyne's criteria (1) and the DSM-5 criteria (21): a refusal to attend school resulting in school absenteeism for a period of at least 1 month; the presence of an anxiety disorder as defined by the DSM-5 (excluding obsessive-compulsive disorder or post-traumatic stress disorder) associated with a negative emotional experience; the absence of conduct disorder; and parental awareness of the whereabouts of the child when not in class. As the collection of ethnic data is forbidden by law in France, we did not ask the participants to disclose their self-identified race and ethnicity. However, we deliberately ensured a diverse recruitment dash–including immigrants and children of immigrants–through purposive sampling, in order to explore both cross-cultural experiences and experiences of racism.

All psychiatrists in the four child psychiatry departments were informed of the study and were asked to screen their patients for inclusion. All school refusers are typically expected to be referred to child psychiatry departments. However, school refusers from underrepresented minorities (such as immigrants and ethnic minorities), may not be identified as such and therefore experience barriers in access to child and adolescent psychiatry departments. Consequently, our population of interest was expected to represent a small percentage of the patients experiencing school refusal in these four departments. Participants' psychiatrists offered them the opportunity to participate in the study on a voluntary basis without compensation. Oral and written information was provided, and oral consent was obtained at the beginning of the interview. The location of the interview was decided with the participants according to their convenience (i.e., child psychiatry department, public place, home). No relationship existed between the participants and the interviewer. The interview included sensitizing questions about: the pathway to school refusal (beginning and course); the participants' understanding of school refusal; their experiences at school (relationships with others, academic performance, experiences of stigmatization or racism), their expectations about their own future; their understanding of parental expectations; their experience of mental health care; their family's history of immigration and the languages spoken at home.

### Sample

In 2019, 16 semi-structured interviews lasting 45 to 90 min were conducted by two researchers (CB and LB) with adolescents and young adults ages 16 to 21 (see Table 1). Because the population of interest is an underrepresented minority among the patients experiencing school refusal in child and adolescent psychiatry departments, the process of identifying and recruiting participants lasted one full year. All patients who were approached about the study agreed to participate. The participants, including immigrants, children of immigrants, and French citizens whose parents were born in the French Caribbean Islands, were representative of minority ethnic groups in France. Their origins reflected the colonial past of France (Algeria, Mali, Morocco, Mauritania) and Great Britain (Ghana, Egypt), the French slave trade in the Caribbean (Guadeloupe, Martinique), intra-European migration (Portugal, Italy, Kosovo), and immigration from the Global South (Turkey, Chile, Colombia, Sri Lanka, Pakistan).

**Table 1:** Characteristics of the population.

**Participants (Pseudonyms)**	**Gender**	**Age**	**Place of birth (PB)**	**Mother PB**	**Father PB**	School attendance^*^
Merlin	M	20	France	France	Kosovo (parents born in Albania)	No
Leila	F	18	France	Algeria	Algeria	No
Dalla	F	17	France	Mali born in Senegal	Mali	No
Léa	F	18	France	France	Italy	No
Joseph	M	21	France	Colombia	Colombia	Yes
Ana	F	16	France	France/Spain	Portugal	No
Michel	M	18	France	France	Martinique (French Caribbean islands)	No
Akash	M	18	France	Sri Lanka	Sri Lanka	No
Amina	F	18	Egypt	Egypt	Egypt	No
Najib	M	19	Algeria	Algeria	Algeria	No
Said	M	16	France	Algeria	Moroccan born in Algeria	No
Joss	M	18	France	France	Chile	Yes
Damia	F	16	Turkey	Turkey	Turkey	No
Mariam	F	18	France	France	Pakistan	Yes
Emma	F	16	France	Guadeloupe (French Caribbean islands)	Ghana	No
Jimmy	M	16	France	Martinique (French Caribbean islands)	Mauritania	Yes

* *Participant has resumed partial or complete school attendance at the time of the interview*.

### Data Analysis

The interviews were recorded, transcribed, and anonymized. Two researchers (CB and LB) independently analyzed all the interviews using Interpretative Phenomenological Analysis (22), whose phenomenological nature invites the researcher to describe in detail the individual experience based on the participant's own formulation of it and the meaning he/she attributes to his/her experience (23). Thus, phenomenological analysis allows a better understanding of individual experience by placing the subject in the position of expert (22). For each interview, we developed detailed codes-full sentences conveying nuances-about the participant's phenomenological experience (perceptions, feelings, meaning making), and wrote a memo summarizing the participant's unique experience. The codes and memos were discussed with the research group (LB, CB, IT, MRM). As in other inductive methods, we did not need to define an exact number of respondents before the research began. The data were coded to generate categories, which in turn were validated through constant comparisons as new interviews were done. Thus, data analysis, further sampling, and theoretical development continued simultaneously until reaching theoretical sufficiency (24). This study is part of the larger project “From school refusal to psychiatric follow-up”, which received a favorable opinion from the Inserm Ethical Evaluation Committee (IRB00003888).

## Results

All participants were ashamed of themselves or of their parents, and all of them experienced feelings of loss (of a group of peers, or of a social status). As their phenomenological experiences were similar, we built a common framework reflecting the feelings of shame, and the perceptions of loss shared by all participants. We divided our findings into three broad domains: (1) in search of a lost identity; (2) suspending autonomy and empowerment; and (3) challenges integrating into the host society. These three main domains comprised six main themes and several sub-themes that logically fit together with one another. In Table 2 we summarize these six main themes. In this section, we use quotes conveying nuances that may not be reflected in the broader themes, and thus enrich our understanding of the participants' individual experiences.

**Table 2:** Domains, themes and sample quotes.

**Domain and themes**	**Sample quotes**
**I. In search of a lost identity**
Straying from the path	“I can't explain it anyway, I went from all to nothing and I never thought I would get to this point” (Leila).
Becoming a stranger to oneself	“I don't even know who I am anymore, I've changed too much, I don't know how I would react to certain situations, I feel like I'm lying to myself” (Akash).
**II. Suspending autonomy and empowerment**
Experiences of parental pressure	“In the Middle East, to be successful, there are two jobs. Ideally either an engineer or a doctor. [Leaving medicine] was an atomic bomb. I was the hope of generations” (Amina).
Distancing oneself from familial roots	“[My father] has a strong accent. It bothers me. When he speaks in Arabic in front of my friends, I don't know, it's embarrassing. I'm ashamed of him” (Said).
**III. Challenges integrating into the host society**
Wanting to keep the peace	“Everyone needs a group of peers, not necessarily friends, but a group they can relate to. A group reassures you, a group makes you feel that you exist in society. When you are not part of society you feel a bit rejected” (Najib).
Envisioning hope: a cross-cultural integration into society	“What I would like to study is something where my Spanish language is an advantage” (Joseph).

### In Search of a Lost Identity

All our participants had been surprised by the onset of their school refusal. Only two of them mentioned a traumatic event leading them to avoid school (i.e., the death of a relative). All the other participants did not recall any specific cause and experienced their behavior as unsettling their sense of identity.

#### Straying From the Path

##### Previously Identifying as a Successful and Ambitious Student

Most of the participants described themselves as dedicated and successful students, loving school and learning: “I was very fulfilled, I loved learning new things” (Michel). The participants' academic ambitions were reflected in their strategic choices: asking teachers for additional help and guidance, attending study groups, choosing selective high school classes—“the hardest thing” (Akash)—or applying to boarding school in hopes of securing better studying conditions than at home. “[My brothers] are noisy and the boarding school, was going to help me to work better” (Dalla). A pervasive belief was that acceptance into a selective class—such as ones offering a scientific curriculum (“S”) as preparation for admission to selective colleges—was the only way to achieve academic success: “I can't do less than the S, there is nothing less than the S, I want to do the S and I will do the S” (Mariam).

##### Being Stunned by One's Own Actions

The participants, who described themselves as model students, perceived their own refusal to go to school as incomprehensible, a source of amazement: “I don't know what happened. [...] I still don't know” (Amina). The participants related their experiences in the passive mode. Like spectators, they could not make sense of the succession of events they described. First came a malaise, which was not limited to academics for most participants. Then a mental block, an inability to go to class, overwhelming their will and their attempts to exercise control: “There is something that prevents me from going there...” (Dalla).

##### Being in Great Pain

For the participants, school refusal could happen to anyone without warning or a reason. Even though the period of suffering at school could last several weeks or months, the decision to stop going to school seemed to happen suddenly: “I can't explain it anyway, I went from all to nothing and I never thought I would get to this point” (Leila). Once they stopped going to school, participants spent their days at home doing little. They might have, at first, read or played video games, but this eventually stopped and time became suspended. The feeling of being useless, of being a burden to others, arose, revealing depressive symptoms: “I'm useless in the world” (Ana). Some participants reported having dark thoughts or suicidal ideation. Some acted on them; two had engaged in self-injury behavior and one had attempted suicide: “It was a week before my midterms, I attempted suicide” (Michel).

#### Becoming a Stranger to Oneself

##### No Longer Recognizing Yourself

Participants found that they no longer recognized themselves and felt disconnected from themselves. “Just the thought of going to class made me anxious. It was really like, this is not me! I don't know, it was like another person coming into my body” (Leila). For some, this disintegration of a coherent identity continued several months after they began refusing to go to school. “I don't even know who I am anymore, I've changed too much, I don't know how I would react to certain situations, I feel like I'm lying to myself” (Akash).

##### Feeling Ashamed of Oneself

Incomplete homework assignments and academic struggles triggered feelings of shame: “I didn't turn it in on time, I didn't come to class because I was too ashamed of not having finished” (Michel). Finally, participants felt ashamed because they had, according to them, “fallen ill”. This shame was so great that they could not talk about it, not even with health care professionals: “I was ashamed to have stopped, I was ashamed to have gotten sick” (Amina). Others thought they were a disgrace to their immediate family, their relatives, and their community because of their “failures”: “When I stopped going to school, it was an absolute shame for my father. He is the Italian immigrant who absolutely wants his children to succeed” (Léa). Many participants were afraid of the future because, according to them, the only way to obtain a status is to succeed in school: “If I don't study, I won't have a career, what will I do later?” (Jimmy).

### Suspending Autonomy and Empowerment

#### Experiences of Parental Pressure

##### A Duty to Succeed

Many of the study participants represented, in their view, their parents' “hope” for academic and professional success. They had one duty—succeed in school: “In the Middle East, to be successful, there are two jobs. Ideally either an engineer or a doctor. [Leaving medicine] was an atomic bomb. I was the hope of generations” (Amina). The difficulties entailed in immigration and relocation were a source of academic pressure that demanded academic success: “They have sacrificed for me, doing jobs they don't like, for their son's future, so I have to give back, doing the best I can, at school” (Joseph). When participants felt that they were no longer able to fulfill their parents' dreams of academic success, a sense of guilt arose. The participants felt obligated to repay their parents by working or costing them as little as possible so that they would no longer be a financial burden: “Even when I went to school, I didn't ask for anything, but now that's one more reason. It's a waste of money” (Akash).

##### Not Being Understood by Your Loved Ones

Participants found that their families, friends, and school administrations did not understand their school refusal, increasing their sense of isolation: “My parents, they don't understand [...] Nobody understands, nobody can understand” (Said). Parental misunderstanding was sometimes expressed through strong reactions, such as shouting or threats to force the child to attend class, and in other cases, participants' parents did not force them to go: “[My mother] didn't force me to go to school” (Mariam).

#### Distancing Oneself From Familial Roots

##### Not Knowing Their Family's Story

Many participants did not have a detailed knowledge of their parents' lives, including their immigration story: “[My father] was probably an adult. Under what conditions, I couldn't tell you” (Mariam). Most participants could name the reasons for immigration or relocation: fleeing a political regime, wanting a better education for their children. Some participants did not mention or were not interested in their parents' immigration: “I don't really care about all that. Since I never asked them too much, and they probably said it but I must have forgotten” (Akash).

##### The Lack of a Shared Language

Despite the multiplicity of languages encountered in their homes, not all participants could find a common language with their families. Indeed, many participants did not fully master their parents' native tongue, and many had parents who were unable to speak French or had difficulty doing so. This created barriers to communication and understanding: “When the psychologist talked to my parents, there was a translator. I don't speak Tamil as well as them. And at the Tamil psychic, there were words I didn't understand” (Akash). Participants seemed to keep their parents' culture separate from life outside the family environment. Almost all participants used or were surrounded by a language other than French, which they did not use in academic settings.

##### Being Ashamed of Your Family

The notion of “shame” was very present during the interviews. Some participants were ashamed of their parents' academic career: “I saw her report cards and I said, ‘But Mom, aren't you ashamed of yourself?”' (Ana, laughing). Others were ashamed of their parents' language or behavior: “[My father] has a strong accent. It bothers me. When he speaks in Arabic in front of my friends, I don't know, it's embarrassing. I'm ashamed of him” (Said). The children of migrant parents were the only participants ashamed of their parents' accents. This subjective experience was part of a broader feeling of shame of one's parents shared by both migrant children and children whose parents were French. Strikingly, the participants overall had attained a lower level of education than their parents. Indeed, the college degrees of parents who arrived in France as adults were often not recognized, and their current jobs were less prestigious than the positions they would have had in their country of origin. Nevertheless, through school refusal, some participants reconnected with their parents or siblings academic or professional struggles. “I am sure that in my family we are not designed to go to school!” (Ana). They found themselves sharing the burden of finding their way without the security of studies.

##### The Loss of Parental Authority

Along with the challenges of self-identification or situating themselves in relation to their parents, some participants described growing up without parental guidance: “It was music that educated me. Not my parents” (Joss). Others identified with and attributed their upbringing to an uncle, a brother, a sister, who were seen as role models and were often idealized: “I wish I had a father like my uncle” (Said). Most participants felt that they have had to manage on their own while also refusing help from their parents. This was particularly true in the case of academics; participants described their parents being unable to help with coursework or provide sound advice regarding their academic careers: “I have no one to help me at home” (Amina). At times, participants observed a role reversal, with parents asking for help from their children: “When [my parents] have a letter to write, they are going to ask my sister. They are not going to do it themselves” (Said).

### Challenges Integrating Into the Host Society

#### Wanting to Keep the Peace

##### Continuing School at All Costs

A painful period, of varying length, separated the onset of the disorder and the complete cessation of going to school. During this period, participants forced themselves to go to school and implemented strategies to cope: going to the school nurse's office, being near a window in class. In spite of headaches, vomiting, crying, sleep disturbances, anxiety attacks, and feelings of dying, they would go back: “I insisted, I continued to go [...], but I still had the lump in my stomach, always, always, all the time” (Dalla). When participants would leave school before the end of the school day, they did not always return home and did not necessarily inform their parents: “I would wait for my parents to leave the apartment, and then I would go home” (Joseph).

##### Denying Racism at School

During the interviews, the topic of racism at times came up. None of the participants said that they had experienced it at school, and none thought of it as a possible cause for their school refusal. In contrast, two of them reported that their parents may have been victims of racism: “She went to a school, but they didn't like her, my mother, because she was Spanish. There was racism in that school” (Ana). However, some participants point out that their foreign origins can be the butt of “jokes”: “At school I was told, ‘But Ana, why be smart? You're Portuguese, you'll end up as a house cleaner”' (Ana).

##### The Fear of Marginalization

According to some participants, the integration in the host country was so important, that their success may have generated the envy of neighbors and acquaintances from their parents' homeland. Leila wondered if she contracted the evil eye in Algeria when neighbors were invited to celebrate her school achievements: “Many people came to my house in Algeria, like 500 people. And I don't know, maybe there were people with bad intentions” (Leila). Many participants mentioned the loss of a group, of a social milieu, concurrent with the start of school refusal. Participants reported that school absenteeism prevented them from feeling “normal” and belonging to the society: “Everyone needs a group of peers, not necessarily friends, but a group they can relate to. A group reassures you, a group makes you feel that you exist in society. When you are not part of society you feel a bit rejected” (Najib).

#### Envisioning Hope: A Cross-Cultural Integration Into Society

At the time of the interview, only four participants had overcome their school refusal, and returned partially or completely to school. They did not differ from the others regarding age, sex, or origin (see Table 1).

##### Finding Support in Their Parents' Culture

Some participants found within their family, their community, or their culture some relief and answers. Cross-cultural discussions enabled them to find different ways to give meaning to their distress, to navigate it and treat it. It also enabled young people to see how their parents allowed themselves to value or reject certain traditions of their country of birth. “I feel better, since I came back from Algeria. Before leaving, I saw imams, they made me drink six liters of water and vomit afterwards. But my cousin […] told me “you shouldn't see an imam, but a person who deals with [evil eye]. You have to heal evil with evil”… Like witchcraft, stuff like that. But my parents are totally against that.” (Leila). Participants were able to seek the help of experts themselves or accept it when their parents offered it. “My parents are so worried that they took me to see a Sri Lankan psychic. He told me that if we could get through this, I would find something I liked, for my studies. And that when I was twenty-three, I was going to get married (laughs). And that it's the girl I'm going to meet that's going to make me change (laughs). But I don't want to believe that, I don't know what to think. It's absurd, but he also described me well. That I'm the kind of person who does things for others. […] If he was a quack, how would he know all this? [...] Talking to a psychic is… weird. But he was saying a lot of true things” (Akash).

##### Envisioning One's Place in Society

Among the services offered, participants benefited from inpatient hospitalizations or intensive outpatient programs. These arrangements allowed participants to leave home, meet other teenagers, and find support among peers even if their challenges were different. “The intensive outpatient program allowed me to get away from home a bit” (Said). Several participants went back to school, often in fields not previously considered or at different institutions such as the Innovative High School (Pôle Innovant Lycéen, PIL), a pilot program to support absent students as they resume high school: “I was doing what they call a PIL. It was great!” (Joseph). Participants seemed to rediscover their cultural resources when they were getting better and on the path to returning to school: “What I would like to study is something where my Spanish language is an advantage” (Joseph). Several participants got involved in their communities and participated in activities where they received recognition and praise for their cross-cultural skills: “I have joined the choir and I teach them Coptic” (Amina).

## Discussion

According to Kearney and Benoit, challenges experienced by students with school attendance problems are exacerbated by disparities in socioeconomic status, childhood adversities, family structure, and neighborhood-level factors (25). Kearney and Benoit argue that advocacy with respect to underrepresented youth with school attendance problems should involve culturally competent care, such as considering family views of the education system, reducing the research-to-practice gap, integrating care across systems (i.e., primary care, education, justice), and developing clinical strategies targeted to specific, high-risk, intersected groups (25). Indeed, cultural and language misunderstandings and racist biases can lead to youth assigned pejorative labels of truancy (18) and caregivers inaccurately viewed as inaccessible, pathological, and unskilled (17). Moreover, young people experiencing high levels of emotional distress can attend school but be absent from the classroom (i.e., remaining in the hallways or playground) (26), leading to punitive rather than restorative care. Our participants experience the intersection of challenges related to school refusal, migration, racism, socioeconomic status, and cultural difference. The distinctive impact of each of these factors on their mental health could not be assessed in this exploratory qualitative study. Nevertheless, their individual experiences may help design culturally competent care for students and families from immigrant and ethnic minority groups.

Previous research on migration have shown that the child, in a cross-cultural context, can experience social and cultural challenges, both those of his parents (i.e., immigration, language barriers) and sometimes his own. Moro (27) describes three periods of vulnerability for the immigrant child: early parent-child interactions (between 0 and 1 year of age), entry into schooling (around 6 to 8 years of age), and early adolescence (between 14 and 16 years of age). Immigration and relocation involve the individual's descendants, and the experience of immigration trauma can be transmitted to the children, whether in the form of a sometimes idealized account or of painful unspoken words (28). Whether it is forced or desired, immigration and relocation can be traumatic, but this trauma will not necessarily have pathological effects; rather, it can also lead parents to model resilience and strength in face of adversity (29). The school environment is not a world linked to the family's culture of origin, and for these children in a cross-cultural situation, entering this space can sometimes be brutal, experienced as exclusion or even violence (30). Although our participants did not report being the target of racist behavior at school, the racism their parents experienced at school remained a painful memory for them. This finding suggests that school might be a place associated with perceptions of suffering, prejudice, and misunderstanding (18), either based on a lived experience or on a transgenerational memory.

In this study, participants described their school refusal as an experience of identity loss. They identified themselves as model students who loved school, which has since become a place associated with painful emotions. Previously, their desire for academic success seemed to be a personal aspiration. But it now appears as a mandate of success imposed by external factors. Faced with their failure, our participants try to make up for their parents' investment; the notion of debt appears, as does that of shame. In this study, shame is present in the family environment (the world inside), but also at school (the world outside). Participants also experience a loss of group membership. They are no longer part of their former group of friends, and this uprooting echoes the literature immigration: leaving the group to which we belong to find ourselves alone in a place where no one is waiting for us (31). Participants describe themselves as strangers to themselves, their families, and their peers. This finding suggests that school refusal may be exacerbated by the intersection of the psychic reorganizations of adolescence and cross-cultural integration. The desire for autonomy and insertion into social life may be challenging when the norms of society appear different–if not opposed–to the ones of the family.

### Suspending Cross-Cultural Integration: The Inside/Outside Divide

Psychological research on adolescence suggests that a first step in becoming autonomous from one's parents is the psychic separation from them, that is, the fact of thinking for oneself (32). This developmental stage appeared particularly hindered for our participants, unable both to attend school and to make sense of their refusal. School refusal is usually associated with underlying mental illness such as generalized anxiety disorder, depression, or social phobia. Nevertheless, Catheline argued that it can also at times reflect an avoidance of autonomous thinking (33). Moro et al. argue that the process of individuation is more complex in cross-cultural situations. It is around the time of the entry into kindergarten that the young child must first separate himself or herself from the family environment (the world of the inside) to find a place in the school environment (the world of the outside and the foreign). In a cross-cultural situation, the child experiences a cleavage, where filiation (parental transmission) and affiliation (belonging to a group) are dissociated (27). In adolescence, a period of identity reconstruction, the subject must integrate his filiation and affiliations. When filiation and affiliations are sources of conflict, the adolescent in a cross-cultural situation may experience difficulties navigating the desire to become autonomous from his or her parents and turn to other objects of investment. It is not an easy task to break certain links with one's culture without wanting to abandon it.

Thus, the ties that adolescent immigrants have to their families and their countries of origin are sometimes ambivalent, sometimes conflicting (34, 35). Moreover, in a cross-cultural situation, the adolescent does not always find figures with whom to identify in the host country, either in his or her peer group or in society. By refusing school and remaining at home, the participants in our study may temporarily avoid the challenge of cross-cultural integration (16), and remain in a single cultural envelope, that of the family. The participants are burdened by the expectations of the different worlds in which they grew up, and their school refusal may reveal their difficulties in processing “*the different influences that cross them and in assuming a multiple-identity construction”* (16).

In this study, participants who expressed a positive projection of the future had begun psychotherapy and thus began a period of self-reflection. Acknowledging their cross-cultural skills and the transgenerational impact of their parents' traumatic experiences (immigration, racism, school dropout), may have helped them overcome their difficulties. In the context of school refusal in immigrant and ethnic minority groups, such a psychotherapy may allow students to weave or reweave healthy connections with the school and maintain comforting connections with the parental culture while accepting the emotional weight of transgenerational suffering. Previous work suggests that, to engage in this process, the adolescent should feel authorized to compare the cultures and behaviors of the people around him or her, and that criticism and fears verbalized should be welcomed and validated (16). Thus, we believe that psychotherapy addressing the specific experience of adolescents with school refusal from underrepresented and intersected groups, should be designed and evaluated. Such inclusive and cross-cultural care already exists for migrant populations. It is provided by a trained therapist or, preferably, by a trained team composed of professionals from various ethnic and cultural backgrounds, or even in a specific setting such as a transcultural group consultation (30). A transcultural group setting enables the patient to think about his or her blending by comparing his or her individual experience with the knowledge of the team members.

### Suspending the Desire for Autonomy: Shame

In this study, few participants knew the immigration history of their parents. For some of them, there was no common language mastered by all members of the family, which may have resulted in obstacles to communication between parents and adolescents. These findings can be discussed considering the literature on migration. Indeed, identity is rooted in part in a family history and family narrative. Having a foundational story allows one to develop a narrative for oneself. However, the transmission of history mostly occurs through the language of the parents, and sometimes parents are discouraged from speaking to their children in their native language. Also, according to Mansouri (36) the terms used to designate young immigrants and members of ethnic minority groups in France predestine them to be cut off from their filiation: “children of immigration” or “from immigration” as if their genealogy began with immigration (37). Immigrant parents bring a cultural heritage with them when they immigrate and act as representatives of that culture. In the host country, they take on the status of “immigrants in the process of integration” in hopes that their child will become a full-fledged member of the host society (30). However, once well-integrated into the host society, the child may appear as a foreigner in the eyes of his or her parents, as they may also want their child to conform to values and norms from the culture of origin. The adolescent is faced with a dilemma: fitting into the host society while remaining a good representative of his culture of origin.

In this study, academic failure was perceived by some participants as family traits. The attempt of our participants to legitimize their parents' lack of academic success was visible through contradicting attitudes: they were ashamed of their parents' poor academic records, while requesting their help to resume school. Thus, refusing school may have enabled them to reintegrate into the family, whereas academic success separated them from their parents. This finding can be discussed considering the literature on migration and social mobility. The desire for a better life for their children, drive parents to relocate and face years if not decades of precarity (31). Consequently, the “duty to succeed” may be a burden on the shoulders of adolescents, as immigrant parents see their child as the reason for their sacrifice (17). The debt to their parents is “paid” by their successful integration into the host society, in particular by their academic success. But academic success may also be perilous for a good student, as it may involve a change in social class. Gaulejac argues that “every individual who changes social class experiences a more or less intense conflict between his inherited identity, the original identity conferred on him by his family environment, and his acquired identity, the one he constructs in the course of his trajectory” (38). One becomes a stranger in one's own home by changing class because this change requires an act of mourning, of unbinding. Bourdieu uses the term *habitus* to describe the norms specific to each social class, the product of the whole biographical experience of an individual (39); the *habitus* is an integral part of the individual. When the change of class takes place between two social groups marked by the historical domination of one over the other, one will be confronted with an invalidation, a devaluation of the behaviors, *habitus*, and values of the “inferior” classes. Social mobility requires a disincorporation of the original *habitus* and a reincorporation of new *habitus* (38).

Most participants in this study perceived themselves as industrious, self-motivated, and high achieving students. They experienced school refusal as a failure and a shame to themselves, to their parents, to their community, and to society. These findings can be discussed considering the literature on academic success. According to MacInnis et al., academic success may trigger an inferiority complex, *impostor syndrome*, defined as “feeling like a fraud who does not belong” (40). One defense mechanism against it is industriousness. Impostor syndrome is increasingly studied in students from minority groups (ethnic, socio-economic, sexual orientation, gender) and is conceptualized as a trauma resulting from micro-aggressions and repeated rejections (41–45). Intersected groups such as immigrants, and ethnic minorities, may develop impostor syndrome because of racism, rejection, and socio-economic difficulties. When experiencing school refusal, these participants may have felt that their imposture was revealed: the “good student” had disappeared and been replaced by the “child of immigrants”.

Several participants had parents born in former French colonies. These participants repeated the word “shame” when referring to their parents' language, culture, and habits. This finding can be discussed considering post-colonial studies. In France, some immigrants face another intersected challenge: the asymmetrical relations between the host country and countries of origin (46). Powerful historical ties, such as colonization or war, also shape cultural representations of “superior” and “inferior” civilizations. Our participants' shame suggests that they may have internalized the prejudice against the social or cultural “inferiority” of their parents.

Taken together, these findings suggest that academic success may create a paradox for these young people: if they succeed, they will become strangers to their families as they change social class and – for some of them – become representants of a country that has oppressed the one of their parents; if they fail, they will dash their parents' hopes and dreams. Thus, considering that “reasonable parental effort” is a specific criterion for school refusal (1) may oversimplify the situation of families with little confidence in their ability to navigate the school system and faced with contradictory attitudes of their child (ambition, blame, shame, impostor syndrome) (17).

### Suspending Integration Into Society: Loneliness

Most of our participants appeared afraid of the judgment of others and had experienced stigma and rejection, but they nevertheless lamented the loss of a group and imagined their peers moving forward without them. This finding suggests that school refusal may confront these adolescents with a blow to their identity formation. Interactionist approaches in social sciences have shown that our identity is a multi-layered, changing, and relational experience, as a result of the outside world's perceptions of us, which are then reflected back onto us: we learn to see ourselves through the eyes of others (47). This mirroring process starts during childhood, within the family and continues at school. In adolescence, the group allows identification with others and with different social roles. As Kestemberg points out “the adolescent only exists through the eyes of others” (48). The literature on migration and racism may also be an interesting framework to discuss our participants' experiences, as they belong to an intersected group. Camilleri proposes three identity-based strategies for immigrants when they are faced with a devaluation, an attack on their self-image (49). The first is to avoid conflict by not seeing oneself as the target of a deprecation. This may be the strategy of our participants, when they report the “humor” of their peers, minimizing their derogatory remarks and refusing to interpret them as racist behaviors. The second strategy consists of confronting the devaluation, and the third allows the construction of a critical identity, where the individual accepts some negative judgments and rejects others. This third strategy allows for integration, in fact; by accepting certain negative judgments, we make our own cultural expectations that were previously unknown to us. Thus, one adopts certain criteria from the host culture and retains others from the culture of the homeland. Of these three strategies, only the first one seemed present in the experiences of our participants.

In this study, our participants experienced feelings of social isolation and worthlessness. Not belonging to a peer group was compared by participants to a rejection from society, an experience that their parents may well have faced when arriving in the host society. This finding suggests that the loss of the group may be particularly significant for adolescents in cross-cultural settings, and this hypothesis could be explored in further studies. On a theoretical perspective, Sayad (31) proposes the feeling of “lasting provisional” in the immigrant that characterizes his or her condition. Indeed, while the immigrant has committed to the host country and the idea of staying there, he or she lives there as if the relocation was temporary. The immigrant finds himself torn between two countries: “on the one hand, the exclusion from the host society which, to varying degrees, affects all immigrants and, on the other hand, the break that is not only spatial from the native land” (31). Adolescents of immigrant parents may be told that they are not French, and in their country of origin they may be told the opposite. Thus, our participants may wonder about where they belong, and where they are desired.

Some of the young people in this study have benefited from therapeutic workshops or groups for adolescents of the same age. One of the functions of these groups may be that the adolescents find crucial moments of doing “nothing” before or after the group, in interstitial places, or outside the host institution. Having in mind only the pleasure of being able to meet themselves and others and knowing that they are accepted are a first step toward an integration into social life. Thus, the effectiveness of group therapies where the adolescents rediscover the value of relationships and can share their experiences, might be worth assessing in underrepresented minority groups.

### Limits and Perspectives

We acknowledge several limitations. This exploratory research is based on a small sample from a single country, and therefore we cannot generalize the experience of our participants, limiting the strength of recommendations for future practice. Several points could not be addressed in this work, notably reflections on how to help teachers in these situations, and how to improve collaboration between the care providers and the school (18), as well as the work that can be done with the adolescent's family.

The participants in this study experienced the intersection of generic issues faced by migrant young people and of issues faced by students presenting with school attendance problems. However, it is not possible in qualitative research to assess the relative impact of each factor (migration, school absenteeism) neither on their school trajectories, nor on their subjective experiences (i.e., shame). Assessing distinctive causalities would be possible in further studies using a longitudinal cohort design. Meanwhile, criteria to differentiate the types of school absenteeism should be used with caution in cross-cultural situations, and their validity should be further explored in immigrant and ethnic minority groups.

Among other treatments, the psychotherapies recommended for school refusal are individual cognitive-behavioral, family, and multifamily therapies (50–52). Future randomized controlled trials could assess the efficiency of a transcultural, trauma-informed and inclusive psychotherapy program for school refusal. Similarly, therapeutic workshops and peer groups for school refusal could be evaluated, to address the fear of marginalization with activities supporting self-esteem and a sense of belonging in all members of the group. To model diversity and inclusivity, such interventions should be designed and delivered by a team of care providers from various cultural and ethnic background.

## Conclusion

In this qualitative study, participants from immigrant and ethnic minority groups experiencing school refusal report the perception of the loss of a group, the shame they feel toward their families and themselves, and the fear of social marginalization. This clinical presentation may challenge the diagnostic criteria for school refusal and contribute to misdiagnosis and reduced access to care for students from underrepresented minorities and intersected groups. Along with assessing parents' customs, culture, immigration history, and experiences of adversity to improve screening and referral to care (17), and in addition to other recommended treatments, transcultural therapies for school refusal should be considered and evaluated to help these students make sense of their experience and increase their sense of belonging, while altogether reducing their academic anxieties.

## Data Availability Statement

The raw data supporting the conclusions of this article will be made available by the authors upon request, without undue reservation.

## Ethics Statement

The studies involving human participants were reviewed and approved by Inserm Ethical Evaluation Committee (IRB00003888). Written informed consent from the participants' legal guardian/next of kin was not required to participate in this study in accordance with the national legislation and the institutional requirements.

## Author Contributions

LB designed the study. CB and LB interviewed the 16 adolescents and young adults and independently analyzed the data. CB, IT, MM, and LB discussed the analysis and results. CB, IT, and LB wrote the manuscript. All authors reviewed the manuscript.

## Funding

This work is supported by the French National Institute of Health and Medical Research (Inserm), and by the Thellie Foundation project improving access to care for school refusal and QUA*Lab*, the Qualitative and Mixed Methods Lab, a collaboration between the the Yale Child Study Center (New Haven, CT), and Inserm, the Institut National de la Santé et de la Recherche Médicale (Paris, France).

## Conflict of Interest

The authors declare that the research was conducted in the absence of any commercial or financial relationships that could be construed as a potential conflict of interest.

## Publisher's Note

All claims expressed in this article are solely those of the authors and do not necessarily represent those of their affiliated organizations, or those of the publisher, the editors and the reviewers. Any product that may be evaluated in this article, or claim that may be made by its manufacturer, is not guaranteed or endorsed by the publisher.
